# Introductory Editorial

**Published:** 2008-03-07

**Authors:** Karna Dev Bardhan

**Affiliations:** Consultant Physician and Gastroenterologist, Rotherham General Hospital, Rotherham, South Yorkshire, U.K., Honorary Professor, Section of Medicine and Pharmacology, University of Sheffield, Sheffield, U.K.

I am pleased to announce the launch of *Clinical Medicine: Case Reports*—a new peer reviewed open access journal published by Libertas Academica. *Clinical Medicine: Case Reports* is an international, open access, peer reviewed journal which considers case reports in all areas of clinical medicine which advance general medical knowledge.

Case reports are not only of academic interest across a wide readership but are also of immediate relevance to practicing clinicians. The majority of doctors across the world practise medicine, and without doubt most have at some time come across “memorable patients” from whom they (and possibly their colleagues as well) have learnt. Many yearn to share their experience; *Clinical Medicine: Case Reports* now offers the ideal vehicle. Reading case reports commonly triggers a variety of memories, emotions and ideas from personal practice and experience. This I feel is a major difference from the research studies published in traditional medical journals: these may or may not influence practice tomorrow whereas case reports can impact today.

Busy clinicians who are prepared to present their patient’s case in an educational forum or informally are sometimes reluctant to write about their experience as they feel “intimidated” by what they perceive as the effort involved in transforming bedside clinical discussions into a cogent and coherent paper. Yet the typical case report does *not* differ greatly from that of cases presented during Ward Rounds, Group Discussions or in Grand Rounds. Simply ask: “What have I learnt from my patient?” “*Lots*”. “Will this information be of interest to others?” “*Yes*”. If so, then try and put the message across directly and in brief. Effort? Yes. Rewards to oneself? Far greater!

This new journal will be competing head-on with a number of existing subscription-based journals, from a position of strength. This is because a journal focusing on case reports is much needed, all its articles will be accessible without any access boundaries to all internet users throughout the world, and anyone can contribute, not only those in major institutions. Thus across the world everyone can contribute and we can learn from each other.

*Clinical Medicine: Case Reports* is published exclusively online. Articles will follow a consistent format so that the visual impact will be high and equal to that of the best hard-copy publications. In contrast to paper-based journals, however, the electronic format allows the full use of digital technologies and permits the inclusion of large data sets, from field and laboratory studies, links to other web pages, animations, slide shows, video clips and unlimited colour, all at no additional charge. Open access means that all articles are freely available to all, worldwide, and at no cost to the reader. Authors retain copyright of their work and can grant anyone the right to reproduce and disseminate it, provided that it is correctly cited and no errors are introduced, under the Creative Commons “CC-BY” licence.

In hard-copy journals, the costs of publication are met by subscriptions, paid by the reader. In *Clinical Medicine: Case Reports*, as in other open access journals, these costs are borne by the author in the form of a publication processing fee. Many grant-awarding bodies recognise the value of open access publishing by allowing their funds to be used for PPFs. Fee waivers and discounts are available on a case-by-case basis, and we shall make every effort to ensure that lack of funds does not impede the overall objective of publishing the best science, irrespective of authorship or country of origin.

I do not foresee that open access, online journals will totally replace the traditional print format in the immediate future, although this may be an increasing trend with time. I am certain, however, that the benefits of online publication, and the extra opportunities that digital technologies give to authors, will be increasingly recognised. Open access is of huge benefit to the clinicians working in institutions around the world where their institutional libraries are unable to afford subscription fees for a full range of journals or indeed in an environment without a library: the internet can be a great equaliser!

I expect that *Clinical Medicine: Case Reports* will attract manuscripts of the highest quality, which are of the greatest possible benefit to readers. After submission, peer review is undertaken by at least two leading experts in the area of the manuscript. Importantly we will at all times strive to encourage and to be supportive. Try us!

For further information on what we hope will be an exciting and highly useful new journal, please click on the “about this journal” link on the journal’s web page.

I look forward to hearing from you.

